# Supplementary material for: Prognostic Value of Salivary Biochemical Indicators in Primary Resectable Breast Cancer
Source: Metabolites. 2022 Jun 16;12(6):552. doi: 10.3390/metabo12060552 (PMC9227854; doi:10.3390/metabo12060552)
Supplement: Supplementary file 1 [file metabolites-12-00552-s001.zip › metabolites-1757937-supplementary.pdf]

Table S1. Biochemical composition of the saliva of patients with primary resectable breast cancer and the control group

№	Indicator	Control Group, n=298	Breast Cancer, n=355	p-value
1	pH	6.49 [6.30; 6.72]	6.48 [6.25; 6.71]	0.8210
2	Calcium, mmol/L	1.27 [1.03; 1.55]	1.24 [0.86; 1.65]	0.6910
3	Phosphorus, mmol/L	4.36 [3.31; 5.85]	4.61 [3.52; 5.94]	0.4550
4	Sodium, mmol/L	8.4 [5.6; 12.1]	8.1 [4.7; 13.0]	0.3636
5	Potassium, mmol/L	10.8 [8.8; 14.2]	11.1 [8.1; 14.3]	0.6745
6	Chlorides, mmol/L	25.5 [20.7; 31.4]	25.5 [20.0; 31.8]	0.3858
7	Magnesium, mmol/L	0.296 [0.241; 0.356]	0.29 [0.22; 0.38]	0.7497
8	NO, µmol/L	22.8 [13.2; 36.8]	29.4 [17.4; 43.7]	0.0010
9	Protein, mg/mL	1.08 [0.65; 1.70]	0.64 [0.38; 1.08]	0.0000
10	Urea, mmol/L	6.66 [4.36; 9.13]	9.89 [6.50; 13.94]	0.0000
11	Uric acid, µmol/L	85.9 [34.4; 144.5]	58.4 [24.2; 127.2]	0.0130
12	Lactic acid, mmol/L	2.32 [1.41; 3.62]	2.33 [1.59; 3.38]	0.2907
13	Pyruvic acid, µmol/L	12.99 [9.56; 18.14]	14.71 [10.29; 19.61]	0.0855
14	Albumin, mg/mL	0.264 [0.175; 0.442]	0.31 [0.18; 0.57]	0.0467
15	α-Aminoacids, mmol/L	4.06 [3.83; 4.32]	4.24 [3.90; 4.81]	0.0000
16	Imidazole compounds, mmol/L	0.303 [0.205; 0.410]	0.281 [0.190; 0.402]	0.0857
17	Sialic acids, mmol/L	0.183 [0.128; 0.293]	0.201 [0.140; 0.275]	0.2520
18	Seromucoids, c.u.	0.091 [0.061; 0.130]	0.100 [0.064; 0.163]	0.0317
19	ALT, U/L	3.85 [2.85; 5.08]	4.15 [3.00; 5.69]	0.0384
20	AST, U/L	5.58 [3.67; 7.50]	6.33 [4.50; 8.83]	0.0026
21	AST/ALT-ratio, c.u.	1.40 [1.13; 1.88]	1.46 [1.15; 1.94]	0.4269
22	LDH, U/L	1101.5 [635.7; 1908.0]	1470.5 [876.7; 2073.0]	0.0003
23	ALP, U/L	60.8 [41.3; 84.7]	71.7 [47.8; 106.5]	0.0017
24	GGT, U/L	20.4 [17.4; 24.4]	23.3 [20.0; 26.5]	0.0000
25	Catalase, nkat/mL	4.58 [3.32; 5.79]	3.80 [2.60; 5.99]	0.0298
26	Superoxide dismutase, c.u.	57.9 [31.6; 113.2]	71.1 [31.6; 147.4]	0.1595
27	α-Amylase, U/L	185.2 [83.5; 384.4]	304.9 [151.3; 602.1]	0.0007
28	Antioxidant activity, mmol/L	1.78 [1.48; 2.28]	1.67 [1.41; 2.10]	0.2647
29	Peroxidase, c.u.	0.400 [0.170; 0.750]	0.440 [0.250; 0.900]	0.2897
30	Diene conjugates, c.u.	3.92 [3.78; 4.07]	3.93 [3.72; 4.13]	0.5179
31	Triene conjugates, c.u.	0.893 [0.818; 0.994]	0.887 [0.789; 1.013]	0.5762
32	Schiff bases, c.u.	0.545 [0.510; 0.576]	0.537 [0.479; 0.671]	0.7031
33	MDA, µmol/L	6.50 [5.73; 7.95]	7.01 [5.73; 8.97]	0.0121
34	MM 280/254, c.u.	0.839 [0.755; 0.948]	0.849 [0.775; 0.948]	0.4935

Note. The description of the sample was made by calculating the median (Me) and interquartile range in the form of the 25th and 75th percentiles [LQ; UQ]. Statistically significant differences between groups are highlighted in red, p<0,05.

Table S2. Biochemical composition of the saliva of patients with primary resectable breast cancer depending on the presence/absence of relapse

№	Indicator	No relapse, n=292	Relapse, n=59	p-value
**1**	pH	6.48 [6.27; 6.71]	6.48 [6.15; 6.75]	0.6130
**2**	Calcium, mmol/L	1.23 [0.92; 1.64]	1.25 [0.82; 1.74]	0.8939
**3**	Phosphorus, mmol/L	4.58 [3.49; 6.01]	4.67 [3.57; 5.62]	0.6799
**4**	Sodium, mmol/L	8.2 [4.7; 13.1]	6.4 [4.9; 10.5]	0.4466
**5**	Potassium, mmol/L	11.4 [8.0; 14.7]	10.3 [8.4; 13.5]	0.4105
**6**	Chlorides, mmol/L	25.7 [20.2; 32.9]	24.9 [19.0; 29.3]	0.1745
**7**	Magnesium, mmol/L	0.29 [0.23; 0.38]	0.28 [0.18; 0.40]	0.5108
**8**	NO, µmol/L	29.5 [17.4; 43.5]	27.9 [19.3; 43.9]	0.5888
**9**	Protein, mg/mL	0.64 [0.38; 1.08]	0.58 [0.38; 1.04]	0.5278
**10**	Urea, mmol/L	9.96 [6.63; 14.04]	9.14 [6.09; 13.14]	0.4795
**11**	Uric acid, µmol/L	60.0 [22.6; 127.8]	57.3 [30.4; 125.0]	0.8382
**12**	Lactic acid, mmol/L	2.30 [1.43; 3.38]	2.73 [1.96; 3.44]	0.1367
**13**	Pyruvic acid, µmol/L	14.71 [10.54; 19.36]	14.22 [9.80; 20.10]	0.8596
**14**	Albumin, mg/mL	0.32 [0.18; 0.58]	0.28 [0.20; 0.41]	0.2346
**15**	α-Aminoacids, mmol/L	4.23 [3.90; 4.79]	4.41 [3.90; 5.10]	0.3243
**16**	Imidazole compounds, mmol/L	0.281 [0.190; 0.410]	0.258 [0.175; 0.357]	0.1800
**17**	Sialic acids, mmol/L	0.201 [0.140; 0.262]	0.220 [0.153; 0.305]	0.1571
**18**	Seromucoids, c.u.	0.099 [0.062; 0.161]	0.106 [0.072; 0.183]	0.2283
**19**	ALT, U/L	4.23 [3.00; 5.69]	4.08 [3.00; 5.38]	0.8830
**20**	AST, U/L	6.33 [4.50; 8.83]	6.50 [4.17; 9.08]	0.8973
**21**	AST/ALT-ratio, c.u.	1.45 [1.13; 1.94]	1.57 [1.22; 1.91]	0.4319
**22**	LDH, U/L	1477.0 [876.7; 2073.0]	1420.0 [767.3; 2101.0]	0.8322
**23**	ALP, U/L	76.1 [48.9; 108.7]	60.8 [47.8; 78.2]	0.0274
**24**	GGT, U/L	23.4 [20.4; 26.6]	22.3 [19.1; 26.3]	0.2418
**25**	Catalase, nkat/mL	3.80 [2.6; 6.02]	3.81 [2.53; 5.83]	0.6184
**26**	Superoxide dismutase, c.u.	71.1 [31.6; 160.5]	65.8 [42.1; 115.8]	0.6407
**27**	α-Amylase, U/L	297.2 [134.2; 568.0]	436.9 [194.7; 670.6]	0.1497
**28**	Antioxidant activity, mmol/L	1.67 [1.41; 2.08]	1.78 [1.41; 2.29]	0.3266
**29**	Peroxidase, c.u.	0.470 [0.250; 0.900]	0.380 [0.150; 0.900]	0.2476
**30**	Diene conjugates, c.u.	3.93 [3.72; 4.17]	3.89 [3.70; 4.10]	0.2529
**31**	Triene conjugates, c.u.	0.882 [0.785; 1.010]	0.905 [0.809; 1.016]	0.3150
**32**	Schiff bases, c.u.	0.536 [0.478; 0.664]	0.553 [0.490; 0.688]	0.4480
**33**	MDA, µmol/L	6.92 [5.73; 8.89]	7.52 [5.98; 9.06]	0.2411
**34**	MM 280/254, c.u.	0.847 [0.773; 0.961]	0.857 [0.794; 0.932]	0.5912

Note. The description of the sample was made by calculating the median (Me) and interquartile range in the form of the 25th and 75th percentiles [LQ; UQ]. Statistically significant differences between groups are highlighted in red, p<0,05.

Table S3. Results of univariate Cox proportional hazards regression analysis

№	Indicator	χ^2	β	Standard Error	t-value	p-value
**1**	pH	0.1881	-0.1339	0.3052	-0.4387	0.6609
**2**	Calcium, mmol/L	0.4682	-0.1411	0.2095	-0.6737	0.5005
**3**	Phosphorus, mmol/L	0.0002	0.0007	0.0536	0.0139	0.9889
**4**	Sodium, mmol/L	0.0548	-0.0045	0.0195	-0.2301	0.8180
**5**	Potassium, mmol/L	0.6793	0.0215	0.0256	0.8388	0.4016
**6**	Chlorides, mmol/L	0.3558	-0.0066	0.0115	-0.5780	0.5633
**7**	Magnesium, mmol/L	0.2660	0.5490	1.058	0.5188	0.6039
**8**	NO, µmol/L	0.0251	0.0005	0.0034	0.1606	0.8724
**9**	Protein, mg/mL	0.6972	-0.1384	0.1717	-0.8057	0.4204
**10**	Urea, mmol/L	0.8464	0.0198	0.0212	0.9345	0.3500
**11**	Uric acid, µmol/L	0.0007	-0.0001	0.0013	-0.0263	0.9790
**12**	Lactic acid, mmol/L	0.1221	0.0214	0.0586	0.3652	0.7150
**13**	Pyruvic acid, µmol/L	1.007	-0.0155	0.0160	-0.9656	0.3342
**14**	Albumin, mg/mL	2.017	-0.5810	0.4336	-1.340	0.1803
**15**	α-Aminoacids, mmol/L	0.0012	0.0032	0.0911	0.0351	0.9720
**16**	Imidazole compounds, mmol/L	0.9717	-0.5979	0.6602	-0.9058	0.3651
**17**	Sialic acids, mmol/L	0.1215	0.1932	0.5310	0.3639	0.7159
**18**	Seromucoids, c.u.	0.1551	0.5795	1.452	0.3990	0.6899
**19**	ALT, U/L	2.422	-0.0040	0.0027	-1.476	0.1399
**20**	AST, U/L	3.761	0.0134	0.0056	2.396	0.0166
**21**	AST/ALT-ratio, c.u.	1.372	-0.2243	0.1995	-1.124	0.2608
**22**	LDH, U/L	0.0081	0.0001	0.0001	0.0901	0.9282
**23**	ALP, U/L	6.378	0.0170	0.0048	3.525	0.0004
**24**	GGT, U/L	0.0304	-0.0037	0.0212	-0.1733	0.8625
**25**	Catalase, nkat/mL	0.4194	-0.0001	0.0001	-0.6419	0.5210
**26**	Superoxide dismutase, c.u.	0.1253	-0.0003	0.0009	-0.3463	0.7291
**27**	α-Amylase, U/L	0.8597	0.0004	0.0004	0.9787	0.3278
**28**	Antioxidant activity, mmol/L	0.0135	0.0441	0.3786	0.1164	0.9073
**29**	Peroxidase, c.u.	0.7713	-0.3223	0.3996	-0.8064	0.4200
**30**	Diene conjugates, c.u.	3.361	-0.7207	0.3973	-1.814	0.0697
**31**	Triene conjugates, c.u.	1.422	0.7208	0.5752	1.253	0.2102
**32**	Schiff bases, c.u.	1.602	0.8465	0.6273	1.349	0.1772
**33**	MDA, µmol/L	0.0082	-0.0044	0.0491	-0.903	0.9281
**34**	MM 280/254, c.u.	0.0057	-0.0514	0.6831	-0.0752	0.9400

Table S4. Results of multivariate survival analysis using the Cox regression model for biochemical indicators of saliva only (χ^2 = 14.19, p=0.00266)

Indicator	β	Standard - Error	t-value	p-value
ALP, U/L	0.0229	0.0060	3.802	0.0001
Diene conjugates, c.u.	-0.0063	0.0030	-2.103	0.0355
AST, U/L	-0.5458	0.3833	-1.424	0.1545
